# Secured Nanosynthesis–Deposition Aerosol Process for Composite Thin Films Incorporating Highly Dispersed Nanoparticles

**DOI:** 10.1002/advs.202204929

**Published:** 2022-12-18

**Authors:** Guillaume Carnide, Yohan Champouret, Divyendu Valappil, Constantin Vahlas, Anne‐Françoise Mingotaud, Richard Clergereaux, Myrtil L. Kahn

**Affiliations:** ^1^ LCC CNRS UPR8241 Université de Toulouse 205 route de Narbonne Toulouse 31077 France; ^2^ LAPLACE CNRS UMR5213 Université de Toulouse 118 route de Narbonne Toulouse 31062 France; ^3^ Laboratoire des IMRCP Université de Toulouse CNRS UMR 5623, Université Toulouse III – Paul Sabatier, 118 route de Narbonne Toulouse 31062 France; ^4^ CIRIMAT CNRS UMR5085 Université de Toulouse 4 allée Émile Monso, BP‐44362, Toulouse Cedex 4 Toulouse 31030 France

**Keywords:** coatings, metal oxide, nanoparticles, organometallic chemistry, safe‐by‐design aerosol

## Abstract

Application of nanocomposites in daily life requires not only small nanoparticles (NPs) well dispersed in a matrix, but also a manufacturing process that is mindful of the operator and the environment. Avoiding any exposure to NPs is one such way, and direct liquid reaction‐injection (DLRI) aims to fulfill this need. DLRI is based on the controlled in situ synthesis of NPs from the decomposition of suitable organometallic precursors in conditions that are compatible with a pulsed injection mode of an aerosol into a downstream process. Coupled with low‐pressure plasma, DLRI produces nanocomposite with homogeneously well‐dispersed small nanoparticles that in the particular case of ZnO‐DLC nanocomposite exhibit unique properties. DLRI favorably compares with the direct liquid injection of ex situ formed NPs. The exothermic hydrolysis reaction of the organometallic precursor at the droplet‐gas interface leads to the injection of small and highly dispersed NPs and, consequently, the deposition of fine and controlled distribution in the nanocomposite. The scope of DLRI nanosynthesis has been extended to several metal oxides such as zinc, tin, tungsten, and copper to generalize the concept. Hence, DLRI is an attractive method to synthesize, inject, and deposit nanoparticles and meets the prevention and atom economy requirements of green chemistry.

## Graphical abstract

Direct liquid reaction‐injection (DLRI) is an innovative approach combining the safe‐by‐design in situ synthesis of NPs prior to their direct injection as an aerosol into a downstream process. DLRI is a secured general synthetic methodology adapted to the formation of nanocomposites. Additionally, DLRI meets several contemporary requirements of green chemistry, namely prevention of exposure to NPs and atom economy.

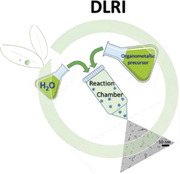

## 1 Introduction

Composite materials that incorporate nanoparticles (NPs) exhibit properties which differ substantially from those of bulk materials.^[^ ^1^, ^2^, ^3^, ^4^, ^5^, ^6^, ^7^ ^]^ NPs dispersed in an organic (e.g., polymer) or an inorganic (e.g., ceramic) matrix form multifunctional nanocomposites. These properties include the matrix, the distribution, concentration, size, and shape of the NPs, as well as the interfaces. Nanocomposites are therefore the subject of intense research in many technological fields and applications.^[^ ^8^, ^9^, ^10^, ^11^, ^12^, ^13^, ^14^, ^15^ ^]^ In particular, nanocomposite thin films, with highly dispersed small NPs (<10 nm) provide multifunctional materials with improved performances.^[^ ^16^ ^]^

However, processing such nanocomposites currently comes with several limitations: i) the general necessity to meet accurate specifications on the NPs such as their size, charge, distribution, etc., ii) the control and reliable reproducibility of the process, and iii) the safety and environmental constraints related to the manipulation of nano‐objects. It is now clearly documented that NPs can adversely affect both the human health and the environment.^[^ ^17^, ^18^, ^19^, ^20^ ^]^ Although some publications report low toxicity for ZnO NPs,^[^ ^21^, ^22^ ^]^ the insecure handling of NPs before any further process implementations comprising the direct application, is currently encountered in classical processes like chemical precipitation, sol‐gel, solvothermal/hydrothermal methods, microemulsion and synthesis from organometallics precursors.^[^ ^23^, ^24^, ^25^, ^26^, ^27^, ^28^ ^]^ The development of processes that avoid contact with NPs remains therefore highly desirable.

On the other hand, Direct Liquid Injection (DLI) is a technology for feeding atomic layer deposition (ALD), chemical vapor deposition (CVD), and plasma enhanced‐chemical vapor deposition (PE‐CVD)^[^ ^29^, ^30^, ^31^ ^]^ with chemical precursors. DLI enables the spraying of NP‐charged suspensions in the form of liquid droplets in dry processes. During aerosol spraying and transport, the aggregation of the preformed NPs is a well‐known limitation. This impedes the access to high dispersion of the NPs, which is desirable when it is incorporated in composite thin films, even when long‐term stable solutions are used. Moreover, the use of additional agents for further stabilization of colloidal solutions is neither atom‐economic nor environmentally friendly. The addition of stabilizing agents and surfactants may also result in the formation of interphases between the NPs and the matrix that deleteriously affects the properties of the final products.^[^ ^13^, ^14^ ^]^

As a response to these constraints, we report herein a secured general synthetic methodology adapted to the formation of nanocomposite thin films. This innovative approach, coined as direct liquid reaction‐injection (DLRI) allows the safe‐by‐design in situ synthesis of NPs prior to their direct injection as an aerosol. In addition, DLRI offers the advantage to be coupled with both dry and wet technologies.^[^ ^32^ ^]^ We illustrate this general synthetic approach through the secured formation of ZnO NPs, and the design of innovative nanocomposites thin films composed of ZnO NPs embedded in a diamond‐like carbon (DLC) matrix.^[^ ^33^ ^]^ The nanocomposite exhibits multifunctional properties, notably a unique water‐repellent behavior. We further generalized the DLRI approach for the design of various oxides, including tin, tungsten, and copper oxides. Additionally, we demonstrate the tolerance of DLRI technology to different solvents or reagents with a potential of preparing industrially relevant nanocomposite thin films.

Thus, DLRI combines technical versatility and safer technology guaranteeing appropriate control of the dispersion of NPs for innovative nanocomposites preparation.

## 2 Results and Discussion

### 2.1 Nanosynthesis Safety Requirement Regarding General DLRI Features and Advantages

The integration of functional NPs within nanocomposite materials has been previously achieved by NP injection, and some aerosol‐assisted methods, which are based on the preformation of suspensions of NPs.^[^ ^34^, ^35^, ^36^ ^]^

For a safe process that avoids any uncontrolled contact of the operator with the NPs, we propose that their synthesis take place immediately prior to their injection in the downstream process. Ideally, this step would occur in a pulsed injection mode, since the time‐off period would enable a controlled formation of the NPs followed by their injection as an aerosol in downstream processes. To its advantage, the in situ synthesis of NPs in the DLRI allows this approach, and prevents any contact with NPs during nanocomposite manufacturing. The DLRI device is comprised of one reaction chamber with two inlets and an outlet. The first inlet is used for the pulsed introduction of the liquid solution containing the organometallic precursor of the NPs diluted in a solvent. The second inlet allows the introduction of a gas phase with a reactant, for example water for the hydrolysis reaction of the organometallic precursor. Both phases react in the mixing chamber. The outlet enables the spraying of the mixture of the formed NPs downstream. The DLRI is then characterized by a set of injection time and frequency parameters (*t*_liq_, *t*_out_, Δ*t*, *f*_liq_ = *f*_out_, see the set‐up detailed in the Supporting Information).

**Scheme 1:**
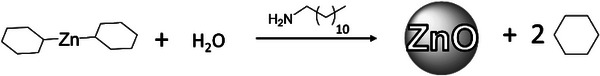
Reaction for ZnO NPs synthesis from the hydrolysis of Zn(Cy)_2_.

The present NP synthesis is specifically designed to be compatible with a direct injection mode and a combined process that allows the manufacture of the composite. As a paradigm of efficiently operating this new DLRI, we synthesize the NPs from organometallic precursor. This approach involves neither a purification step, nor corrosive byproducts, it operates at low temperature, is fast and ensures a quantitative yield of well‐defined NPs.^[^ ^37^, ^38^, ^39^, ^40^ ^]^ The formation of ZnO NPs by this approach is highly appealing as ZnO NPs present wide technological applications due to their unique properties. These include electro‐optical properties,^[^ ^41^, ^42^ ^]^ which can be used in devices such as ultraviolet (UV) light‐emitting diodes (LEDs),^[^ ^42^, ^43^ ^]^ blue luminescent devices or UV lasers^[^ ^44^ ^]^; photo(electro)catalytic water treatment,^[^ ^45^, ^46^, ^47^ ^]^ antibacterial agents,^[^ ^48^, ^49^ ^]^ solar cells,^[^ ^50^, ^51^, ^52^, ^53^, ^54^ ^]^ and others.^[^ ^55^, ^56^ ^]^ Such unique properties require not only the ZnO material to be nanosized, but also to be homogeneously dispersed without agglomeration.^[^ ^57^, ^58^, ^59^ ^]^ We thus chose the synthesis of ZnO NPs using the hydrolysis route of organometallic precursors, and test its potential compatibility with a pulsed injection mode. As a matter of fact, the controlled hydrolysis of dicyclohexyl zinc, Zn(Cy)_2_, in batch reactors is known to produce well‐defined crystalline ZnO NPs of <10 nm in diameter (**Scheme** **1**).^[^ ^60^ ^]^ The fast hydrolysis of Zn(Cy)_2_ solubilized in various organic solvents (THF, pentane, etc.) takes place with one equivalent of water in the presence of dodecylamine (C_12_H_27_N, DDA),^[^ ^61^ ^]^ a suitable ligand for ZnO. Note that the ligand may also play a role on the hydrolysis reaction.^[^ ^62^, ^63^, ^64^ ^]^ The synthesis can be performed at 20—25 °C under atmospheric pressure, which are conditions fully compatible with DLRI. Under these conditions, the organometallic precursor is highly reactive at rates compatible with the DLRI. The device consists of the separate introduction of a liquid pentane solution of Zn(Cy)_2_ and of a reactive gas phase in a carrier inert gas flow (herein, water vapor in argon). The solvent (pentane) was chosen for the formation of DLC films through plasma deposition. Note that the byproducts (Scheme 1, cyclohexane) are also suitable precursors for the matrix.

### 2.2 ZnO NPs Synthesis from DLRI

**Figure 1:**
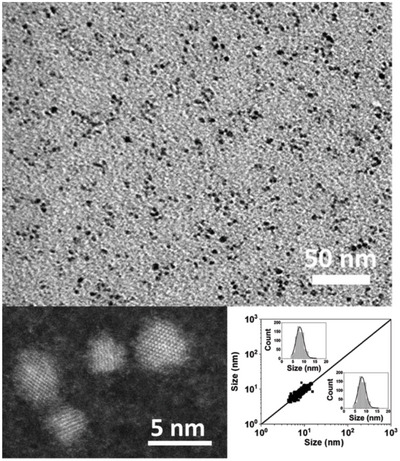
TEM (top), HRTEM images of ZnO NP collected at the exhaust of the DLRI, and the corresponding 2D size plot (bottom right).

We achieved ZnO nanosynthesis by DLRI (15 mL of pentane for 0.4 mmol Zn(Cy)_2_, i.e., 0.027 M, 20°C, 45 min, *t*_liq_ = 5 ms, *t*_out_ = 10 ms, Δ*t* = 2 ms, *f*_liq_ = *f*_out_ = 1 Hz, see details in the Supporting Information). The solution NMR analysis of the aerosol sprayed at the exit of the DLRI proves the complete consumption of Zn(Cy_2_) precursor (Figure S1 in the Supporting Information).^[^ ^65^ ^]^ The transmission electronic microscopy (TEM) images corresponding to the formed NPs (**Figure** **1**), clearly indicate that a single population of isotropic ZnO NPs was obtained, which are characterized by an average diameter of 8.3 ± 1.7 nm. The multidimensional correlation between length and width in a collection of (an)isotropic particles can be assessed by 2D size plot analysis.^[^ ^66^ ^]^ This approach, compared to the more common measurement and statistics of NPs diameter, points out the topological diversity of NPs, and their degree of isotropy.^[^ ^67^ ^]^ Herein, 2D size plot (Figure 1 and Figure S2, Supporting Information) exhibits a “single point” cloud along the diagonal line, which is characteristic of 1) isotropic NPs, i.e., produced by homogenous growth mechanisms in all directions and 2) nonaggregated with only 0.1 equiv. of DDA versus the zinc precursor used as a stabilizing agent.

The high resolution TEM (HRTEM) images revealed crystalline NPs (Figure 1, bottom left, and Fourier Transform (FT) in Figure S3 in the Supporting Information) with typical hexagonal zincite structure. The interplanar distances are measured at 0.285, 0.256, and 0.250 nm, correlating the (100), (002), and (101) interplanar distances expected for würtzite ZnO (0.281, 0.260, and 0.247 nm, respectively). The as‐obtained NPs moreover exhibit optical properties (absorption and emission) characteristic of ZnO (Figure S4, Supporting Information).^[^ ^68^ ^]^

The ZnO NPs are similar whether they are obtained in solution or in DLRI, suggesting that the same mechanism at the molecular level takes place in both cases. Even so the controlled hydrolysis of homoleptic zinc alkyls may involved RZn‐OH and cubane species,^[^ ^69^, ^70^, ^71^ ^]^ it is still a challenging issue for over 150 years.^[^ ^72^ ^]^

Thereby, the organometallic NP synthesis methodology that we adapted herein for ZnO NPs nanosynthesis is fully compatible with a combined direct reaction‐injection process. Moreover, the process promotes the formation of an aerosol consistently populated of small, well‐defined, isotropic and crystalline NPs.

### 2.3 DLRI Process Compared to the Injection of Ex Situ Preformed Colloidal Solutions of NPs

**Figure 2:**
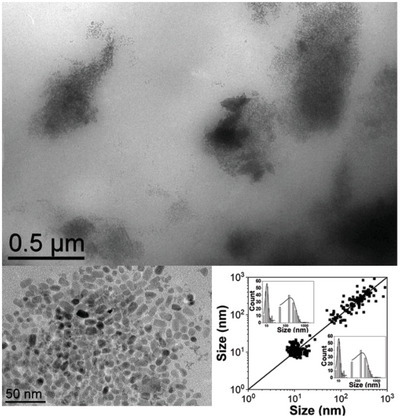
TEM images and the corresponding 2D size plots of the exhaust of ex situ formed ZnO NPs spayed using DLI.

The reported analogous synthesis of ZnO NPs are classically prepared in batch processes in solution using Schlenk tube glassware,^[^ ^60^, ^61^ ^]^ needing at least one equiv. of DDA to achieve the colloidal stabilization in THF solution. We independently synthesized pentane colloidal solutions of ZnO NPs in batch processes following the classical methodology of hydrolysis of an organometallic solution in Schlenk tubes (details on the preparation of ex situ ZnO NPs in the Supporting Information). When using 0.1 equiv. DDA (the same conditions used for the production of NPs by DLRI) we repeatedly observed the formation of large aggregates of more than 100 nm. This indicates a largely insufficient amount of stabilizing agent to prevent ZnO aggregation (see TEM and dynamic light scattering (DLS) analyses detailed in the Supporting Information, Figure S5). In order to form stable colloidal solutions of ZnO NPs in pentane, the ex situ approach requires 150 times more DDA stabilizing agent in solution than for the secured DLRI. This suitable colloidal solution of ex situ formed ZnO NPs was further sprayed with the DLRI as an aerosol on TEM grids. The resulting TEM picture and the corresponding 2D size plots of the exhaust (**Figure** **2**) clearly show the aggregation of NPs (details in the Supporting Information). Take note of the two point clouds present in the 2D size plot in Figure 2 which is characteristic of a strong aggregation of NPs. These results highlight the benefits of DLRI as a secured method of processing NPs for human operators, in the economy of stabilizers, and on the effectiveness of the DLRI aerosol for producing small and dispersed ZnO nanoparticles. To help position the DLRI process with respect to existing technologies, the environmental factor (E‐factor)^[^ ^73^ ^]^ is determined and compared for the production of ZnO NPs based aerosols using DLRI on one hand and DLI of the colloidal solution on the other hand. This simple metric is defined as the ratio of the mass of waste per mass of product. Based on the chemical reaction reported in Scheme 1 and the quantity of DDA involved in both conditions and used to stabilize the nanoparticles, E‐factors of respectively 1.7 and 29.3 were obtained highlighting the relevance of the use of DLRI in a sustainable greener approach.

### 2.4 Chemical and Specific Interfacial Processes Occurring in the DLRI for ZnO NP Growth

Because of the significant difference observed from the DLRI synthesis of ZnO NPs compared to the injection of ex situ preformed colloidal solutions, we assessed the mechanism of this new process, and in particular, how it differs from the ex situ solution‐phase synthesis of NPs and its subsequent DLI. We thus focused on pertinent parameters of interest, which includes the number of particles formed, their aggregation state, and the nature of the aerosol. We initially examined conditions in which the number of NPs formed is similar in DLI and DLRI (for details on this number of NPs, see the Supporting Information). With this parameter established, we were able to evaluate the expected state of agglomeration of the NPs by considering the mass conversion law (see Supporting Information). Aggregated NPs from DLRI would be expected while they were found clearly isolated. This strongly suggested that a decisive specificity of DLRI comes from the downstream aerosol.

**Figure 3:**
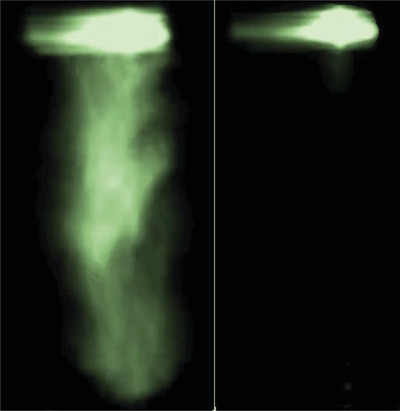
Snapshot of the aerosols produced with DLI (left) and with DLRI (right). The strong upper light halo corresponds to a light reflection on the metal part of the experimental set‐up.

This assumption was experimentally confirmed through light scattering experiments. As a matter of fact, we found that the nature of the aerosol in DLRI and DLI strongly differ from one another. **Figure** **3** illustrates snapshots of movies recorded by a high speed camera and taken 20 ms after the injector aperture (full movie is reported as Movie S1 in the Supporting Information). While a fog of droplets is clearly observed along the aerosol of pentane produced with DLI (left), the exhaust of the DLRI exhibits an extremely low level of scattered light (right). Considering Mie scattering, it means that the number and/or the size of the droplets in the DLRI aerosol are greatly reduced at the exhaust.

**Figure 4:**
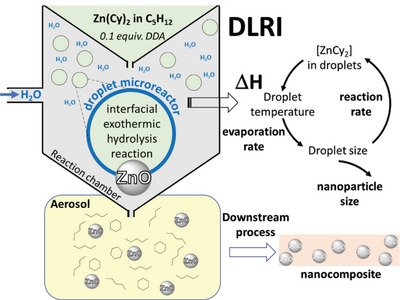
Schematic view of the DLRI process: example of ZnO.

In DLRI, we correlated this behavior with the hydrolysis reaction involving the Zn(Cy)_2_ organometallic precursor. Since water and pentane form an immiscible binary system at room temperature,^[^ ^74^ ^]^ and since the DLRI device is designed to ensure the mixing of the liquid with the carrier gas to obtain an aerosol,^[^ ^75^ ^]^ we can thus safely assume, as schematized in **Figure** **4**, that the hydrolysis reaction takes place at the interface ‐an original growth site‐^[^ ^76^ ^]^ between the liquid droplets containing the zinc precursor and the gas phase containing water. The exothermic hydrolysis induces first a temperature increase, which directly improves pentane evaporation in the reaction chamber and leads, consequently, to a decrease in the droplet size. Therefore, the resulting aerosol at the exhaust of the DLRI exhibits smaller droplets as imaged by light scattering. In addition, pentane evaporation increases the concentration of Zn(Cy)_2_ in the liquid droplet and, at least, at the interface liquid‐gas. Consequently, at the same initial concentration, the smaller the droplets are, the higher the hydrolysis completion is.

The control of the DLRI parameters allows to establish the optimized conditions for ZnO synthesis. For example, for [Zn(Cy)_2_] = 0.025 mol L^−1^, *f*_liq_ = *f*_out_ = 1 Hz, Δ*t* = 2 ms, *t*_out_ = 10 ms, and *t*_liq_ = 2 to 5 ms, the complete consumption of the zinc precursor is evidenced by NMR spectroscopy, while for *t*_liq_ = 10 ms, peaks characteristic of the zinc precursor are observed (Figure S6). The increase in *t*_liq_ corresponds to an increase in the volume of solution introduced into the reaction chamber, i.e., an increase in the quantity of precursor to be hydrolyzed. As the reaction chamber is always saturated with water vapor, this reagent cannot be considered limiting. The reaction time, Δ*t* = 2 ms, then appeared insufficient for the reaction to be complete. As previously discussed, this can be attributed to the number and the size of droplets in the mixing chamber that become larger as *t*_liq_ increases. These two parameters have two correlated consequences: on the one hand, the hydrolysis reaction of the whole precursor is no more possible and on the other hand, the evaporation of the solvent induced by the exothermic reaction will be lower. There is therefore a correlation between the precursor concentration, the DLRI parameters, and the aerosol shape, which will obviously influence the aerosol injected in the downstream process and, consequently, the final nanocomposites.

This DLRI mechanism, favoring small dispersed NP nucleation in microreactor droplets using solvents of various physical‐chemical properties (as evidenced by the similar absorption bands independently of the solvent – Figure S7, Supporting Information) provides a further lever of control. For example, it is possible to modify the aggregation state (see Figure S7 in the Supporting Information).

We, therefore, envisioned this process as potentially suitable for producing nanocomposites with the incorporation of adequately distributed and isolated NPs in various matrices.

### 2.5 DLRI Extension for the Controlled Formation of DLC‐ZnO Nanocomposites

The preparation of NPs using DLRI, which combines in situ nanosynthesis in tandem with nanoinjection, presents the benefit of being “safe‐by‐design” for operators regarding the handling and exposition of NPs. The DLRI can be connected to various dry or wet processes and does not require the use of a vaporization box. The inclusion of the ZnO NPs formed by in situ synthesis from DLRI, in a DLC matrix was achieved by using PE‐CVD as the downstream process. Here, it is directly connected to the shower electrode of a RF‐plasma chamber. The resulting nanocomposite was produced through pulse the NPs embedded in the matrix formed through the electron inelastic collisions with the solvents and, eventually the byproducts.

The Zn(Cy)_2_ organometallic precursor being fully soluble in pentane at the concentration used (0.025 M), pentane was employed as a precursor of DLC matrix from PE‐CVD. Of note, cyclohexane resulting from the hydrolysis reaction (Scheme 1) also contributes to the DLC matrix.

**Figure 5:**
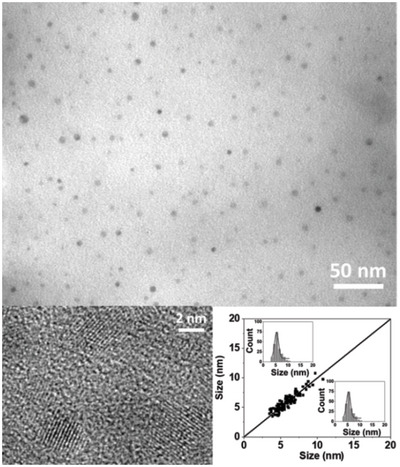
TEM (top), HRTEM (bottom left) images of DLC‐ZnO nanocomposite, and the corresponding 2D size plot.

TEM images of the nanocomposite films formed in the DLRI coupled with PE‐CVD and deposited on a grid are presented in **Figure** **5**. The resulting nanocomposite presents the inclusion of well‐dispersed, nonaggregated small NPs as expected. The 2D size plot analysis (Figure 5, bottom right) clearly indicates the presence of a single population of isotropic NPs with an average diameter of 5.8 ± 1.4 nm (see also Figure S8, Supporting Information), comparable to the population characterized from the exhaust of DLRI aerosol. HRTEM reveals the same highly ordered crystalline structure with interplanar distances that correspond to würtzite ZnO (Figure S9, Supporting Information), confirming that the features of the NPs are not affected by the high‐energy plasma process.

Hence, DLRI coupled with PE‐CVD enables the forming of nanocomposite thin film at room temperature (Figure S10, Supporting Information).^[^ ^77^ ^]^ The resulting DLC‐ZnO thin films exhibit hydrophobic and water‐repellent properties without microtexturation and high mechanical resistance,^[^ ^78^, ^79^, ^80^, ^81^, ^82^, ^83^, ^84^ ^]^ (see Figure S11, Supporting Information, and the full movie reported as Movie S2 in the Supporting Information). This makes their application as antifog, antifouling, or antiicing coatings possible.

### 2.6 Extending DLRI Nanosynthesis–Deposition to other Processes, Matrix, and Metal Oxides

DLRI is compatible for coupling with various dry coating processes such as CVD, ALD,^[^ ^85^ ^]^ and as detailed herein, PE‐CVD. Remarkably, in addition to providing a new and unique ZnO nanosynthesis in different solvents (e.g., pentane, cyclohexane, and toluene), we also demonstrated that ZnO NPs are formed when using liquid reagents such as hexamethyldisiloxane (HMDSO) and tetraethoxysilane (TEOS), two chemical compounds that are very commonly used for the production of functional SiO_2_ matrices (Figure S12, Supporting Information).^[^ ^86^, ^87^, ^88^ ^]^ We thus assumed that –as long as the organometallic precursor is compatible with the matrix precursor molecule(s)–, DLRI is ideally suited to any dry process of nanocomposite formation incorporating NPs, with various matrix of the relevant application.

Considering the mechanism of droplet microreactors above described, the various level of control for DLRI process, and the extensive bibliography of reactive organometallic precursors, the clean hydrolysis of organometallics in DLRI can be generalized to the synthesis of many metal oxide NPs. We further illustrated this concept with the easy formation of copper, tin, and tungsten metal oxides (see Figure S13 and the Supporting Information for details on the preparation and characterization of these various oxides NPs from DLRI). We successfully obtained, as proof of concept, from the hydrolysis of i) {Cu_2_[2,6‐(i)Pr_2_C_6_H_3_N)_2_C(H)]_2_}, highly dispersed crystalline NPs of 2.6 ± 0.6 nm diameter with typical copper oxide structure (Figure S13A, Supporting Information); ii) from [Sn(NMe_2_)_2_]_2_, ≈2 nm size crystalline NPs (Figure S13B, Supporting Information) with interatomic distance characteristic of Sn_3_O_2_(OH)_2_ phase; and from W(NtBu)_2_(NHtBu)_2_, ultrasmall crystalline NPs (*d* >1 nm, Figure S13C, Supporting Information) with interatomic distances characteristic of a tungsten oxide phase.

## 3 Conclusion

We report here an innovative approach, called Direct Liquid Reaction‐Injection (DLRI), which alleviates several nanomaterial processing bottlenecks. DLRI, as a new aerosol‐based method, achieves the in situ chemical synthesis of NPs prior to their injection into a downstream process, paving the way for the formation of a new generation of processes for nanocomposites materials. Herein, the preparation of ZnO NPs from hydrolysis of the organometallic Zn(Cy)_2_ was shown as an ideal way to form a multifunctional nanocomposite by using this safe‐by‐design DLRI process. For example, coupled with a PE‐CVD process downstream, DLRI leads to multifunctional DLC‐ZnO nanocomposite with the inclusion of well‐dispersed, nonaggregated NPs smaller than 10 nm. DLRI is superior to the classical DLI of colloidal solutions, in which, agglomerations of the ex situ formed ZnO NPs are obtained with an aggregate size of over 100 nm. These results show that DLRI is an accurate and robust method that enables the manufacturing of nanocomposites in a safe manner for operators, with atom economy for NP growth stabilizers, and which fulfills some of the requirements for sustainable chemistry.

The nanosynthesis‐deposition process described herein was successfully generalized to various matrixes and other metal oxides, showing the genuine versatility of DLRI. We anticipate that DLRI extends beyond hydrolysis reactions for the formation of metal oxides. For example, metallic or chalcogenide NPs are currently under investigation through hydrogenolysis and sulfidation of organometallic precursors, respectively.

Overall, DLRI provides a simple, economic, and environmentally friendly preparation method, guaranteeing appropriate control of the size, morphology, and dispersion of NPs in a matrix for the preparation of a new generation of nanocomposites with multifunctional properties to explore.

## Conflict of Interest

The authors declare no conflict of interest.

## Supporting information

Supporting Information

Supplemental Movie 1

Supplemental Movie 2

## Data Availability

The data that support the findings of this study are available from the corresponding author upon reasonable request.
