# Supplementary material for: Secured Nanosynthesis–Deposition Aerosol Process for Composite Thin Films Incorporating Highly Dispersed Nanoparticles
Source: Adv Sci (Weinh). 2022 Dec 18;10(5):2204929. doi: 10.1002/advs.202204929 (PMC9929256; doi:10.1002/advs.202204929)
Supplement: Supplementary file 1 — Supporting Information [file ADVS-10-2204929-s001.pdf]

Supporting Information

Secured Nanosynthesis–Deposition Aerosol Process for Composite Thin Films Incorporating Highly Dispersed Nanoparticles

Guillaume Carnide, Yohan Champouret, Divyendu Valappil, Constantin Vahlas, Anne-Françoise Mingotaud, Richard Clergereaux, Myrtil L. Kahn**

CONTENT:

- DLRI set-up **S1**
- ^{1}H NMR spectra of the aerosol for parameters leading to the complete consumption of the $[Zn(Cy_2)]$ precursor (t_{liq} = 5 ms, t_{out} = 10 ms, Δt = 2 ms, f = 1 Hz) **S2**
- 2D size plot associated to the TEM images of ZnO NP collected at the exhaust of the DLRI **S3**
- HRTEM of the ZnO NPs obtained at the exhaust of DLRI **S8**
- Optical properties of the ZnO NP collected at the exhaust of the DLRI **S8**
- Preparation of the ex-situ ZnO NPs **S9**
- DLS analysis **S9**
- Typical TEM images, associated 2D size plot, and correlogramms of ZnO NPs pentane solutions for various quantity of DDA **S10**
- Evolution of the NP average diameters as determined by 2D size plot analyses of TEM images and DLS **S12**
- DLI from ex situ prepared ZnO NPs **S12**
- Mass conversion law, formation of aggregates **S13**
- Estimation of the number of NPs **S13**
- ^{1}H NMR spectra for DLRI conditions where the hydrolysis reaction is not complete (t_{liq} = 10 ms, t_{out} = 2 ms, Δt = 2 ms, f = 1 Hz) **S14**
- Absorption, TEM image, and associated size distribution of ZnO NPs formed using the DLRI with the Zn precursor dissolved in cyclohexane, and toluene **S14**
- Preparation of the nanocomposites using DLRI coupled to PE-CVD downstream process **S15**

- 2D size plot associated to the TEM images of DLC-ZnO nanocomposite obtained by Coupling DLRI with PE-CVD **S15**
- HRTEM of the DLC-ZnO nanocomposite obtained using DLRI coupled with PE-CVD **S16**
- Thickness of DLC-ZnO nanocomposite film *versus* deposition time **S16**
- Wettability properties of DLC-ZnO nanocomposite prepared using DLRI coupled to PE-CVD **S17**
- TEM and HRTEM of the SiO_2-ZnO nanocomposite obtained using DLRI coupled to PE-CVD **S17**
- Preparation of various oxides in DLRI **S18**

DLRI set-up:

The device used was an Atokit injection-atomizer from Kemstream Inc. (Montpellier).[1] It consisted in an injection head comprising a liquid injector, a mixing chamber (where the liquid phase is mixed with the feeding gas) and a output injector for the exhaust (Scheme S1).

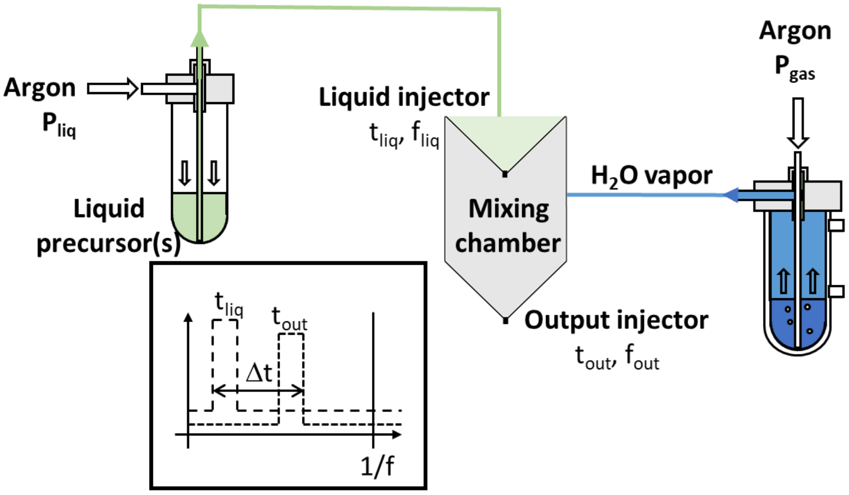

Scheme S1: Experimental set-up. The process aims to synthesize NP from the mixture of liquid and gas phases prior to their injection as an aerosol in the downstream plasma reactor to form nanocomposite thin films.

The device was connected upstream to a tank filled with the liquid phase and the gas phase, both pressurized at fixed pressures (P_{liq} and P_{gas}). The liquid is pulsed injected through the liquid injector during an opening time, t_{liq}, and a frequency, f_{liq}, and mixed with the gas phase in the mixing chamber during a delay, Δt. The aerosol was sprayed in a pulsed mode through the output injector during an opening time, t_{out}, at a frequency, f_{out}, generally set equal to f_{liq}. The device is then characterized by the set of parameters: (t_{liq}, t_{out}, Δt, f_{liq}=f_{out}). Note that the DLRI does not require the use of a drying chamber.

Liquid and gas phases description for ZnO NPs synthesis using DLRI:

The liquid and gas phases consisted respectively of a pentane solution (15 mL) containing [Zn(Cy)$_2$] (0.025 mol.L^{-1}) with 0.1 eq. of dodecylamine (DDA, 0.0025 mol.L^{-1}) used as a ligand and argon bubbling through water. As the chemical compounds used here are highly sensitive to water, the liquid solutions were prepared inside an MBraun glovebox with an argon system. [Zn(Cy)$_2$] (purchased from Nanomeps) and used without any further purification, stored at -20 °C, was weighed and added to a Fisher Porter flask. Pentane dried using an MBraun SPS column - the residual water contents of the solvent being systematically measured by Karl Fischer coulometric titration (Metrohm) - was added, and the solution manually stirred to homogenize it. Finally, DDA (from Sigma-Aldrich) was added to the solution.

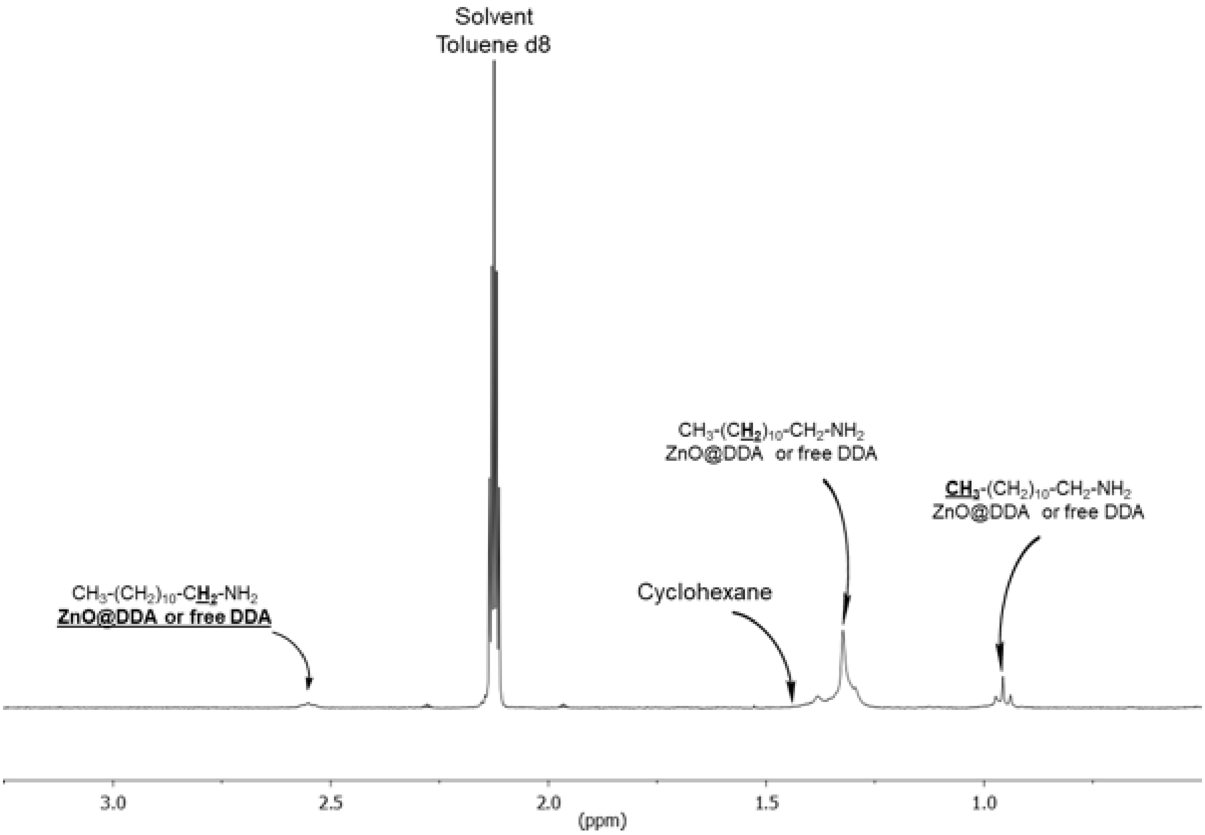

Figure S1. ^{1}H nuclear magnetic resonance (NMR) of the aerosol (t_{liq} = 5 ms, t_{out} = 10 ms, Δt = 2 ms, f = 1 Hz). The spectrum confirms the complete consumption of the [Zn(Cy$_2$)] precursor. Only peaks associated to DDA in interaction with the ZnO NPs or free in solution are observed, as previously reported for the synthesis of the ZnO NPs following the same organometallic approach but in solution.[2]

2D-size plot and multivariate analysis:

2D plot analysis.[3] Each particle is characterized by two sizes, noted D1 and D2, measured on perpendicular axes. These two sizes generally correspond to the length and width of the nano-objects. For each particle and on a same graph, we plot D1 as a function of D2 and also D2 as a function of D1. The aspect ratio of the particle (noted AR) is defined as the ratio between the two lengths AR=D1/D2 (with D1>D2). It is related to a theta angle through the equation AR=tan θ. Higher anisotropy in the shape of a particle corresponds to higher AR value and to a theta angle closer to 90° in the proposed 2D plot. 2D plots present point clouds whose structures can be the result of different sub-populations. To identify these sub-populations, a multivariate analysis was performed with the MIXMOD software (http://mixmod.org) using R package. Apart from the Gaussian character of the probability densities, no assumption about the orientation, shape, and volume of the different sub-populations was made during calculation. The number of sub-populations that composed the point clouds was fixed by the user or chosen numerically thanks to the BIC criteria (Bayesian Information Criterion). Each sub-population was then characterized by the mean of the two studied variables (i.e., short and long axis lengths), as well as the corresponding standard deviations. Additionally, the correlation parameter ρ between both variables can be calculated. The correlation is equal to zero when the two variables are totally independent and equal to 1 when they are affinely related to each other.

MIXMOD software

The main purpose of the MIXMOD software (http ://www.mixmod.org) is to discover group structures in multivariate data sets. It is an exploratory data analysis tool for solving clustering and classification problems. Mathematically speaking, for quantitative multivariate data, the MIXMOD software modelises the probability density of the data $x_i = (x_i^1, \cdots, x_i^d)^T$, $i = 1, \cdots, N$ by a mixture f of K multivariate Gaussian densities $h_1, \cdots, h_K$:

$$f(x_i, K, (\mu_k, p_k, \Sigma_k)_{k=1,\cdots,K}) = \sum_{k=1}^{K} p_k h_k(x_i, \mu_k, \Sigma_k) \qquad (1)$$

where :

$$h_k(x_i, \mu_k, \Sigma_k) = \frac{1}{\sqrt{2\pi}^d \sqrt{|\Sigma_k|}} \exp\left(-\frac{1}{2}(x_i - \mu_k)^T \Sigma_k^{-1} (x_i - \mu_k)\right)$$

h_k is caracterized by a mean vector μ_k and a variance-covariance matrix Σ_k. $|\Sigma_k|$ denotes the determinant of Σ_k. The estimation of the parameters μ_k, p_k, Σ_k for $k = 1, \cdots, K$ is done by an EM (Expectation-Maximization) algorithm. The aim of this algorithm is to find the "best", the "most likely" estimators of the parameters μ_k, p_k, Σ_k for $k = 1, \cdots, K$ that is to say the estimators that maximize the likelihood :

$$\prod_{i=1}^{N} f(x_i, K, (\mu_k, p_k, \Sigma_k)_{k=1,\cdots,K})$$

This algorithm consists in calculating iteratively until convergence :

$$\alpha_{ik}^{(n)} = \frac{p_k^{(n)} h_k(x_i, \mu_k^{(n)}, \Sigma_k^{(n)})}{\sum_{k=1}^{K} p_k^{(n)} h_k(x_i, \mu_k^{(n)}, \Sigma_k^{(n)})}$$

$$\mu_k^{(n+1)} = \left(\sum_{i=1}^{N} x_i \alpha_{ik}^{(n)}\right) / \left(\sum_{i=1}^{N} \alpha_{ik}^{(n)}\right)$$

$$\Sigma_k^{(n+1)} = \left(\sum_{i=1}^{N} \alpha_{ik}^{(n)} (x_i - \mu_k^{(n)})^T (x_i - \mu_k^{(n)})\right) / \left(\sum_{i=1}^{N} \alpha_{ik}^{(n)}\right)$$

$$p_k^{n+1} = \left(\sum_{i=1}^{N} \alpha_{ik}^{(n)}\right) / N$$

When the estimators $\hat{\mu}_k$, $\hat{p}_k$, $\hat{\Sigma}_k$ for $k = 1, \cdots, K$ are found ; each observation x_i is affected to its "most likely" component $\hat{k}_i$:

$$\hat{k}_i = \text{argmax}_k \frac{\hat{p}_k h_k(x_i, \hat{\mu}_k, \hat{\Sigma}_k)}{\sum_{k=1}^{K} \hat{p}_k h_k(x_i, \hat{\mu}_k, \hat{\Sigma}_k)}$$

Thus a classification in K classes is obtained.
For each K we can calculate $\hat{\mu}_k$, $\hat{p}_k$, $\hat{\Sigma}_k$ for $k = 1, \cdots, K$. To estimate K we choose a model (1) that fits well the data but without too many parameters. For that we choose K that minimizes the BIC (Bayesian Information Criterion) criterion :

$$\hat{K} = \text{argmin}_K \text{BIC}(K) = \text{argmin}_K(-2\ln \prod_{i=1}^{N} f(x_i, K, (\hat{\mu}_k, \hat{p}_k, \hat{\Sigma}_k)_{k=1,\cdots,K}) + \nu_K \ln(N))$$

where ν_K is the number of free parameters in the mixture model with K components.

Statistical indices

The mean vector of a data set $x_i = (x_i^1, \cdots, x_i^d)^T$, $i = 1, \cdots, N$ is estimated by

$$\bar{x} = \frac{1}{N} \sum_{i=1}^{N} x_i.$$

The variance is estimated by

$$\hat{\text{Var}}(x) = \frac{1}{N-1} \sum_{i=1}^{N} (x_i - \bar{x})^2$$

The standard deviation is defined as the square root of the variance. The covariance between two real variables x^1 and x^2 is estimated by

$$\hat{\text{Cov}}(x^1, x^2) = \frac{1}{N-1} \sum_{i=1}^{N} (x_i^1 - \bar{x^1})(x_i^2 - \bar{x^2}).$$

The correlation between x^1 and x^2 is estimated by

$$\hat{\rho}(x^1, x^2) = \frac{\hat{\text{Cov}}(x^1, x^2)}{\sqrt{\hat{\text{Var}}(x^1)}\sqrt{\hat{\text{Var}}(x^2)}}$$

The correlation is equal to zero between two independent variables and equal to 1 between two identical variables.

Modified mixmod software

In the modified version of the MIXMOD software we assume that the first component of the mixture (1) is known. Thus the probability density of the data $x_i = (x_i^1, \cdots, x_i^d)^T$, $i = 1, \cdots, N$ is a mixture f of K multivariate Gaussian densities $h_1, \cdots, h_K$:

$$f(x_i, K, (\mu_k, p_k, \Sigma_k)_{k=1,\cdots,K}) = \sum_{k=1}^{K} p_k h_k(x_i, \mu_k, \Sigma_k)$$

where only μ_1 and Σ_1 are known. The other parameters are unknown. The points belonging to the first component are chosen in a 5% confidence interval around μ_1. Then the standard MIXMOD software is used to classify the other points and determine the number of classes.

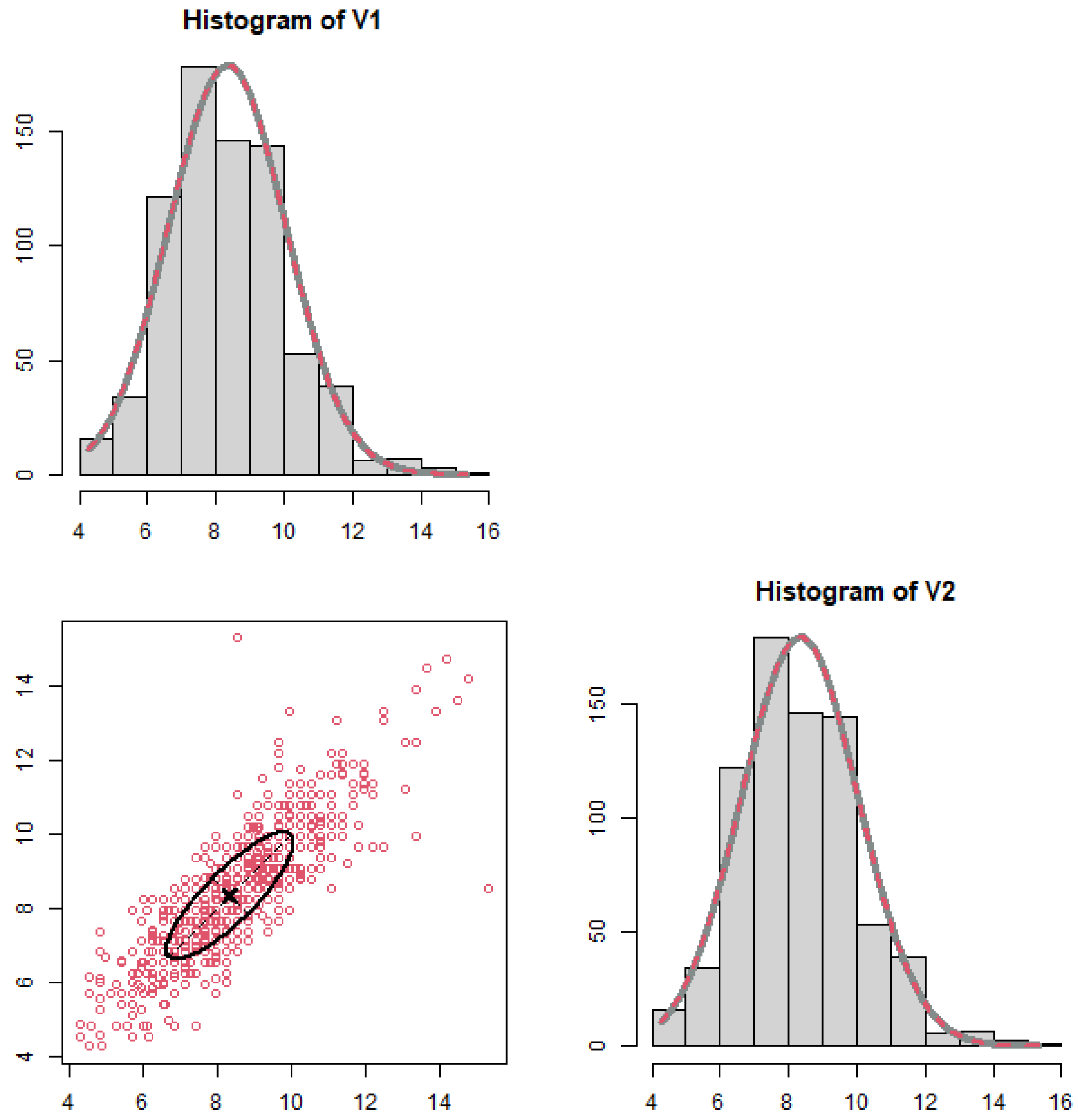

Figure S2. 2D size plot[3] associated with the TEM images of ZnO NP collected at the exhaust of the DLRI. The point cloud lies along the median line, which is characteristic of isotropic NPs. The growth is homogenous in all directions. Multivariate analysis leads to only one population with a mean size value of 8.3 ± 1.7 nm.

2D-size plot shown Figure S2 presents a point cloud along the diagonal of the plot, which is characteristic of isotropic nanoparticles. The multivariate analysis leads to the following results:

```
****************************************
*** MIXMOD Models:
* list =  Gaussian_pk_Lk_Ck
* This list includes only models with free proportions.
****************************************
```

```
* data (limited to a 10x10 matrix) =
      V1    V2
 [1,] 9.234 9.109
 [2,] 10.79 9.091
 [3,] 11.81 9.659
 [4,] 10.23 9.091
 [5,] 7.955 7.955
 [6,] 7.676 6.534
 [7,] 10.79 10.37
 [8,] 7.102 6.962
 [9,] 9.091 9.091
[10,] 6.678 4.974
* ... ...
****************************************
*** MIXMOD Strategy:
* algorithm            =  EM
* number of tries      =  1
* number of iterations =  200
* epsilon              =  0.001
*** Initialization strategy:
* algorithm            =  smallEM
* number of tries      =  10
* number of iterations =  5
* epsilon              =  0.001
* seed                 =  NULL
****************************************


****************************************
*** BEST MODEL OUTPUT:
*** According to the BIC criterion
****************************************
* nbCluster   =  1
* model name  =  Gaussian_pk_Lk_Ck
* criterion   =  BIC(4977.9719)
* likelihood  =  -2472.4358
****************************************
*** Cluster 1
* proportion =  1.0000
* means      =  8.3452 8.3452
* variances  = |     2.9640     2.5065 |
               |     2.5065     2.9640 |
****************************************
```

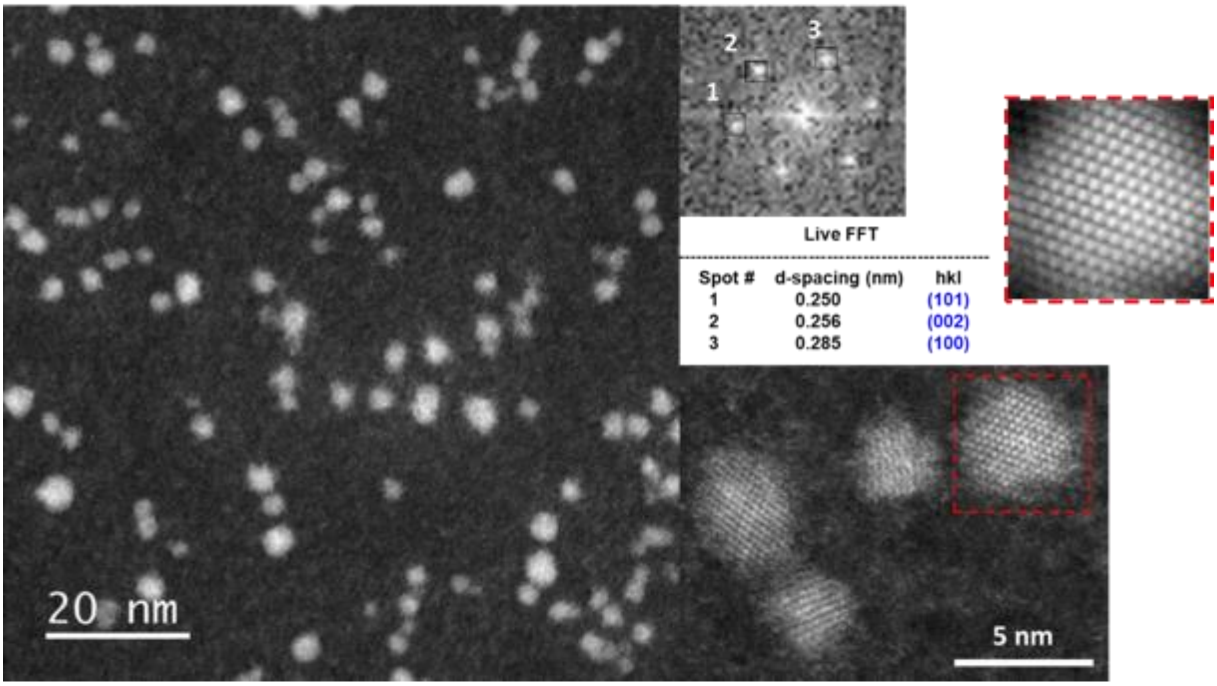

Figure S3. HRTEM of the ZnO NPs obtained using DLRI. The distances measured from the Fourier transform of the HRTEM image are d = 0.285, 0.256, and 0.250 nm, *i.e.*, within the accuracy of the measurements, in very good agreement with the (100), (002), and (101) interplane distances anticipated for wurtzite ZnO (0.281, 0.260, and 0.247 nm, respectively).

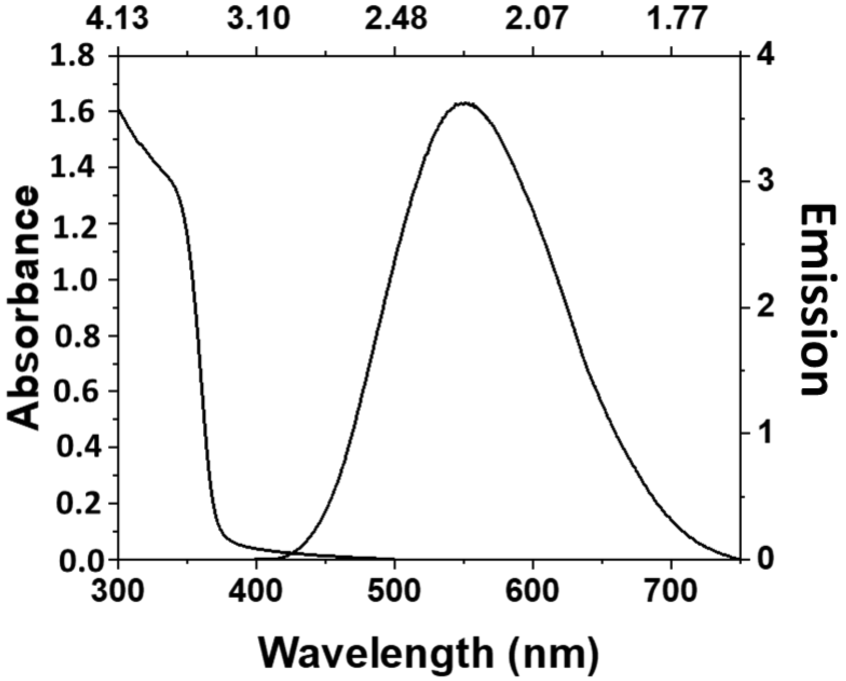

Figure S4. Optical properties of the ZnO NP collected at the exhaust of the DLRI.

Preparation of the *ex-situ* ZnO NPs:

For comparison, colloidal solutions were prepared. It consists of pentane solutions with preformed ZnO NP using DDA as the ligands. All the preparations were carried out inside the glovebox. First, the [Zn(Cy)$_2$] was weighed (86 mg for a concentration of 0.025 mol.L^{-1}) and add to a Schlenk tube glassware. The pentane (15 mL) was added and the solution was manually stirred until a homogenous solution is obtained. Then, different concentration of DDA was made from 0.1 to 20 molar equivalent to the zinc precursor. For this, the DDA was weighed (from 7 mg for 0.1 equiv. to 1.4 g for 20 equiv.) and added to the solution. The solution was manually stirred until a homogenous solution was obtained.

In a Schlenk tube, some dry THF was added and a desired amount of distilled and degassed water added (typically 9.9 mL of dry THF with 0.14 mL of degassed water).

Outside the glovebox, 1 mL of the water-containing THF was added dropwise to the stirred solution of [Zn(Cy)$_2$] and DDA. The solution was left under stirring overnight for complete reaction. The DDA concentration was adjusted from 0.1 to 20 equiv. Colloidal stability was then evaluated by DLS measurements and TEM characterization.

DLS analysis:

Dynamic Light Scattering (DLS), carried out at 25°C on a Malvern (Orsay, France) Zetasizer NanoZS, was used to find optimal colloidal solutions. Solutions were analyzed in triplicate without being filtered in order to characterize the plain samples. Data were analyzed by the general-purpose non-negative least squares (NNLS) method. The typical accuracy for these measurements was 10-20% for systems exhibiting a polydispersity index lower than 0.4. All correlograms were analyzed by a custom-made program named STORMS in order to obtain a more precise characterization of the solutions.[4] This program has been designed with Matlab and enables the fitting of DLS correlograms using different sets of parameters, corresponding to all hypotheses that have to be made during the treatment. Indeed, going from correlograms to size results implies three levels of hypotheses: the first one consisting in the transformation of autocorrelation data to diffusion coefficient, the second one extracting the size of the scattering object from diffusion coefficient depending on its geometry, and finally using a model enabling the transformation of the intensity-relative population to a number-relative one. For each step, STORMS provides the choice of different parameters. For the nano-objects presented here, the protocol used a NNLS fitting, assumed a spherical shape for all objects, and the chosen scattering model was Rayleigh for objects smaller than 100 nm and Mie for larger ones. Figure S5A shows the correlograms obtained from the DLS measurement

of the various ZnO NPs pentane solutions as a function of the equivalent of DDA as stabilizing agent as well as the associated TEM images and the related 2D size plots.

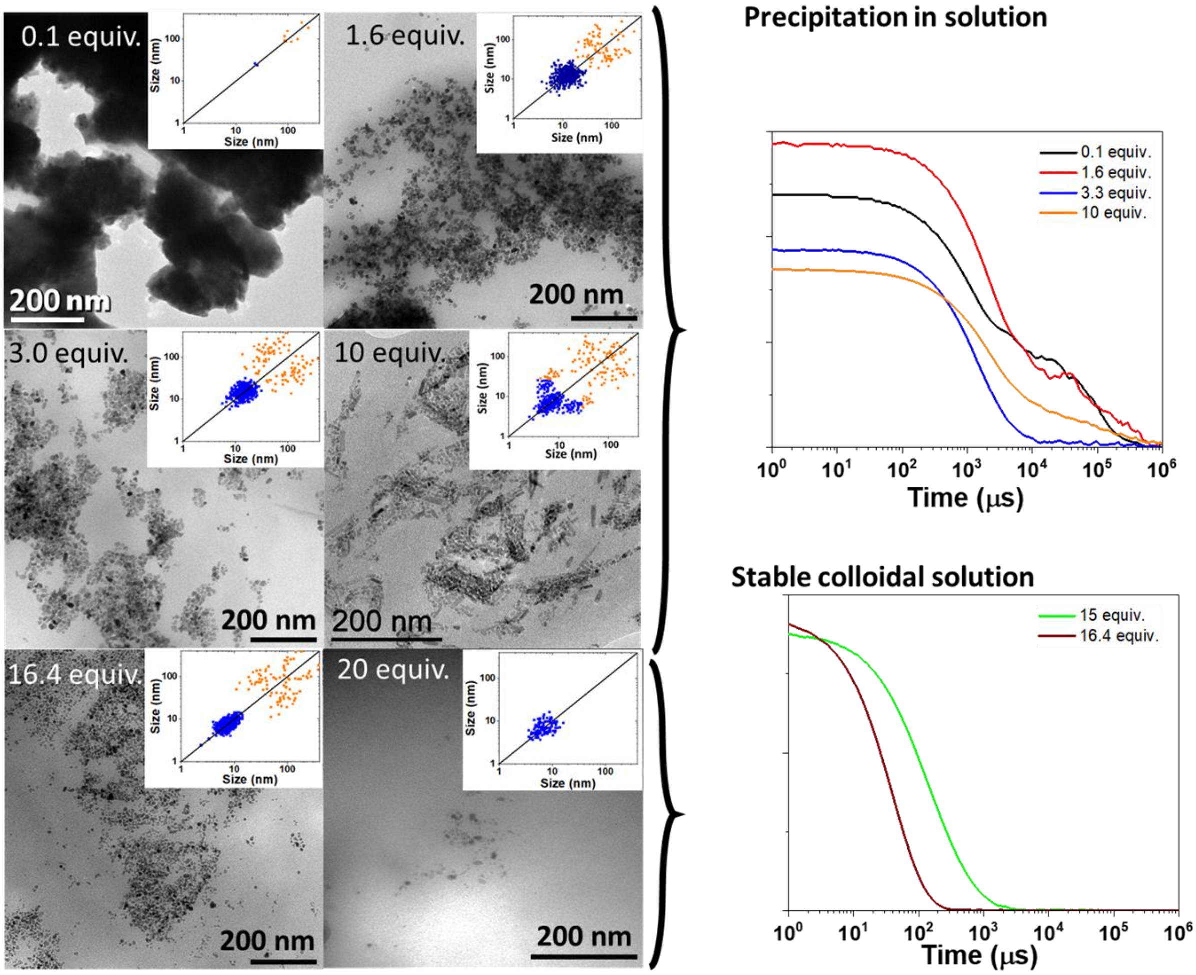

Figure S5A. Typical TEM images, associated 2D size plot,[3] and correlogramms of various ZnO NPs pentane solutions for various quantity of DDA as stabilizing agent. Those experiments were performed in order to find the conditions to have a stable colloidal solution in pentane that can be injected in the plasma chamber for comparison with DLRI.

Typically, 3 types of correlograms are observed: those with low DDA ratios (0.1 and 1.6), which are unexploitable, the ones with medium DDA ratio (3.3 - 10), which start to exhibit a regular correlogram but the reproducibility on the same solution was too bad, sign of a strong instability of the solution, and finally those with high DDA ratio (15 and 16.4) for which a good reproducibility of the correlograms is observed.

The intensity-averaged analysis shows for all samples, multiple populations (see Figure S9B below). The number-averaged analysis reproduces the distribution in 3 types of correlograms already mentioned (see Figure S9C below). The only solutions giving satisfactory results are for ratios 15 and 16.4, but the hydrodynamic diameter measured are far from the size of the

NPs determined from the TEM. There are several possible reasons for this, the most likely being that aggregates much larger than the NPs themselves exist in the solution.

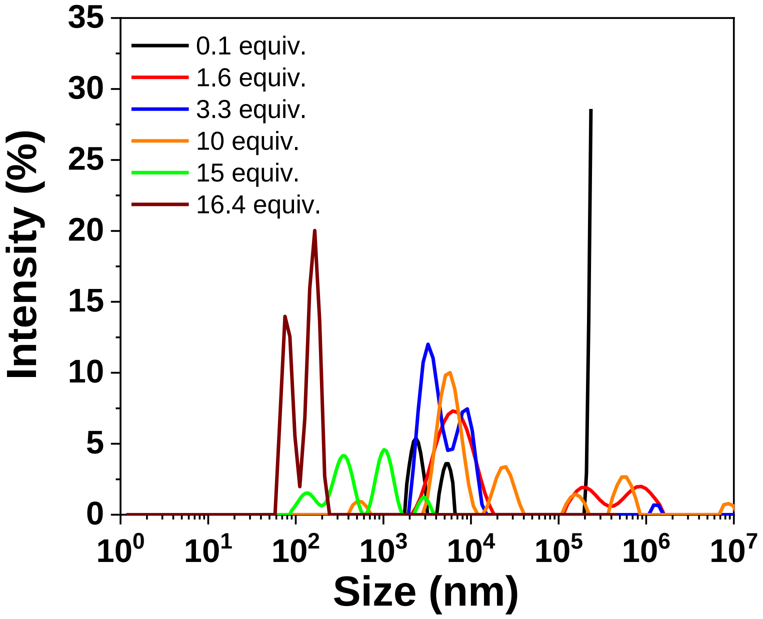

Figure S5B. Intensity-averaged analysis for all samples for which multiple populations is observed.

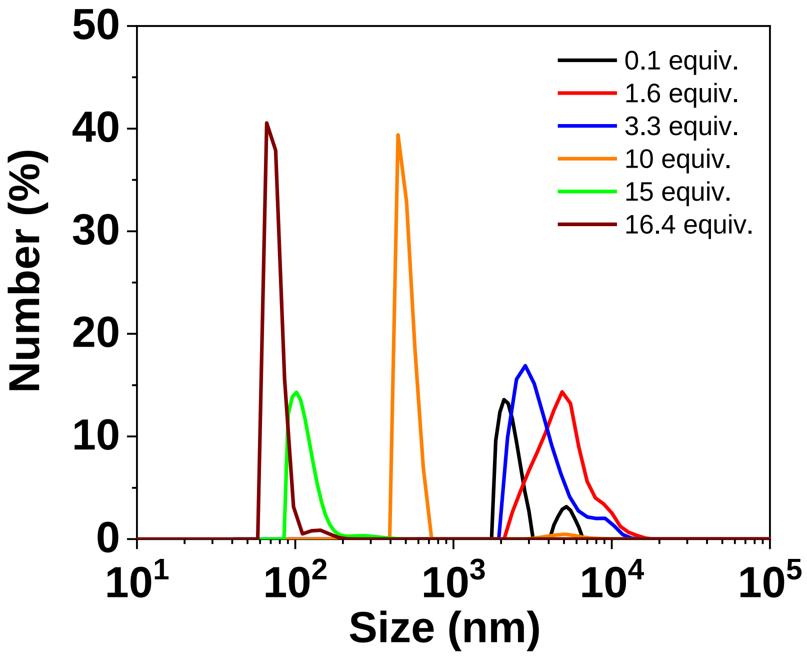

Figure S5C. Number-averaged analysis.

Although comparison of the absolute size values between DLS and microscopy should be made with precaution,[4-5] a similar trend was obtained (Figure S5D). The 2D size plot analyses of TEM images (Figure S5A) correlated with DLS analysis, with increasing DDA

leading to a size decrease of the NPs. Notably, NPs are in this case polydispersed, with a second population *c.a.* 10% formed of anisotropic aggregates.

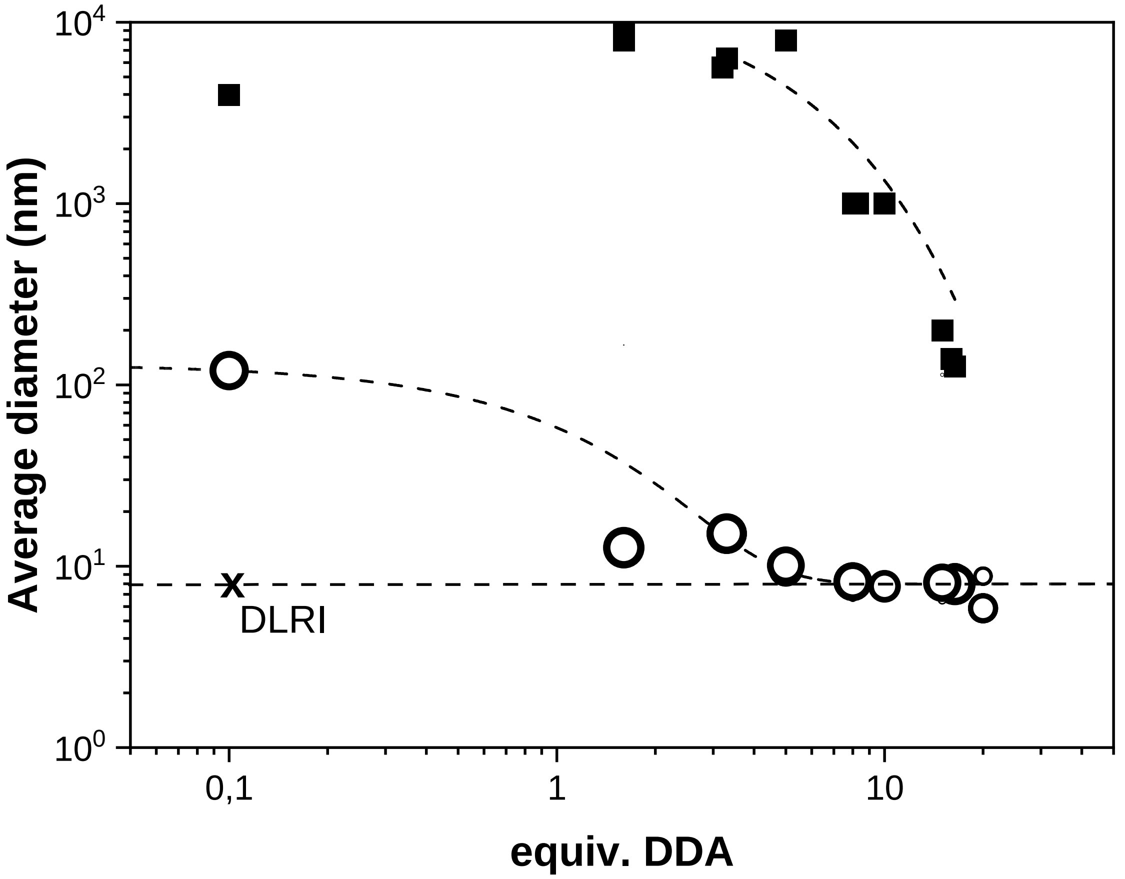

Figure S5D. Evolution of the NP average diameters as determined by 2D size plot analyses of TEM images (circles) and DLS (squares) as a function of the concentration of DDA in a pentane solution. For comparison, the size of ZnO NP formed in DLRI is reported as a cross.

The best conditions determined for *ex-situ* formed NPs obtained a stable colloidal solution of ZnO in pentane were obtained in synthesis employing 15 equiv. or more of DDA as stabilizing agent.

DLI from ex situ prepared ZnO NPs:

The Fisher Porter flask containing the colloidal solution was connected to the DLI device under argon. Aerosol containing NP was sprayed in a home-made glassware allowing were deposited on copper grids for TEM analyses.

Mass conversion law and formation of aggregates

Evaporation of the solvent in the droplets will form aggregates. Considering the mass conversion law, for a given concentration of NPs, the final average aggregate size, D_{AG}, is directly proportional to the initial droplet size, D_d, of the aerosol:

$$D_{AG} = \left(\frac{c_{[Zn(Cy)_2]} \cdot M_{ZnO}}{\rho_{ZnO}}\right)^{1/3} \cdot D_d \qquad \textbf{Eq. 1}$$

In the present experimental conditions, aggregates must be two orders of magnetude smaller than the droplet size with $D_{AG} \approx 0.03 \cdot D_d$. Considering that droplets in aerosol are in the micrometer-size range, ZnO NP should be found aggregated in the few tens to hundreds of nm range as observed in the direct injection of ex situ formed ZnO colloidal solutions (Figure S6a).

Estimation of the number of NPs:

For a given concentration of $c_{[Zn(Cy)_2]}$, the quantity of the organometallic precursor injected per pulse, $N_{[Zn(Cy)_2]/pulse}$, is a function of the liquid flow rate, r_{liq}, *i.e.* of the liquid injection time t_{liq} and frequency f_{liq}:

$$N_{[Zn(Cy)_2]/pulse} = r_{liq} \cdot c_{[Zn(Cy)_2]} \qquad \textbf{Eq. 2}$$

In the appropriate condition for ZnO NPs, 5 mL of liquid solution are injected for 15 min corresponding to a r_{liq} = 5.5 µL.s^{-1}. With $c_{[Zn(Cy)_2]}$ = 0.025 mol.L^{-1}, it corresponds to $N_{[Zn(Cy)_2]/pulse}$ = 2.5·10^{-7} mol/pulse.

Since the reaction is quantitative,[6] one mole of ZnO NPs is produced per mole of [Zn(Cy)$_2$], *i.e.* $N_{[Zn(Cy)_2]/pulse} = N_{ZnO/pulse}$. The theoretical number of ZnO NP produced and injected per pulse, $n_{ZnO/pulse}$, is therefore directly dependent on the liquid flow rate:

$$n_{ZnO/pulse} = N_{[Zn(Cy)_2]/pulse} \cdot \frac{M_{ZnO}}{v_{ZnO\text{-}NP} \cdot \rho_{ZnO}} \qquad \textbf{Eq. 3}$$

where $v_{ZnO\text{-}NP}$, M_{ZnO} and ρ_{ZnO} are, respectively, the volume of a single ZnO NP, its molar mass and its density. It means that for $c_{[Zn(Cy)_2]}$ = 0.025 mol L^{-1} and a homogeneous isotropic shape of NP with the previous average diameter, as observed from the TEM images, DLRI theoretically yields $n_{ZnO/pulse}$ = 1.3 ± 0.0·10^{13} NP/pulse, corresponding to 2.4·10^{18} NP L^{-1} in each droplet in the aerosol. Such value is comparable to the one of the glassware solution as the $ZnCy_2$ concentration is the same and the size of the resulting ZnO nanoparticles comparable.

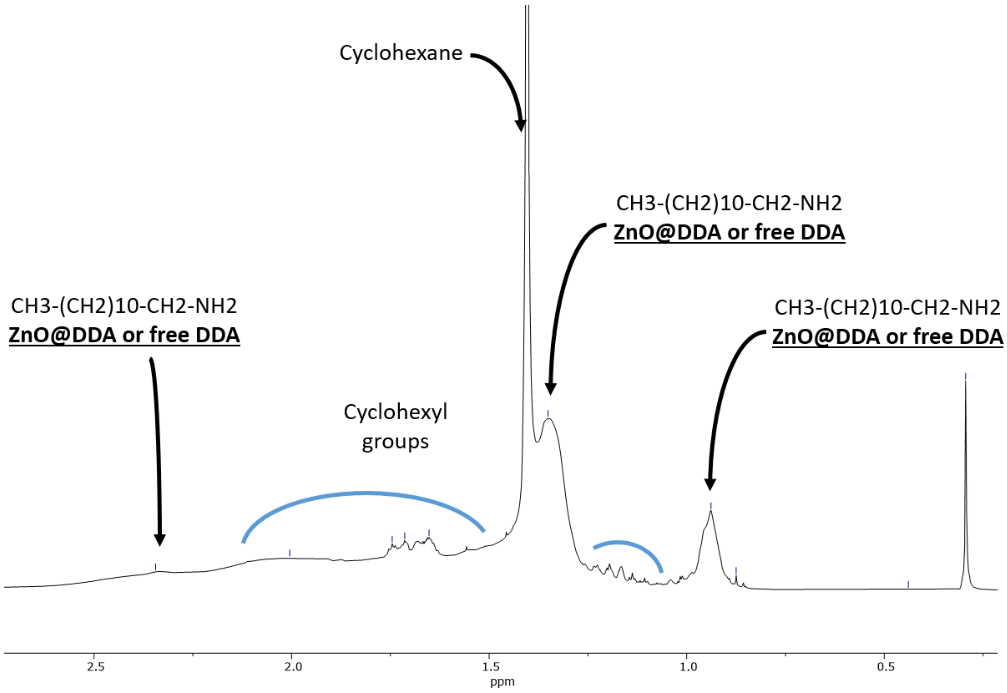

Figure S6. ^{1}H NMR spectra for DLRI conditions where the hydrolysis reaction is not complete (t_{liq} = 10 ms, t_{out} = 10 ms, Δt = 2 ms, f = 1 Hz).

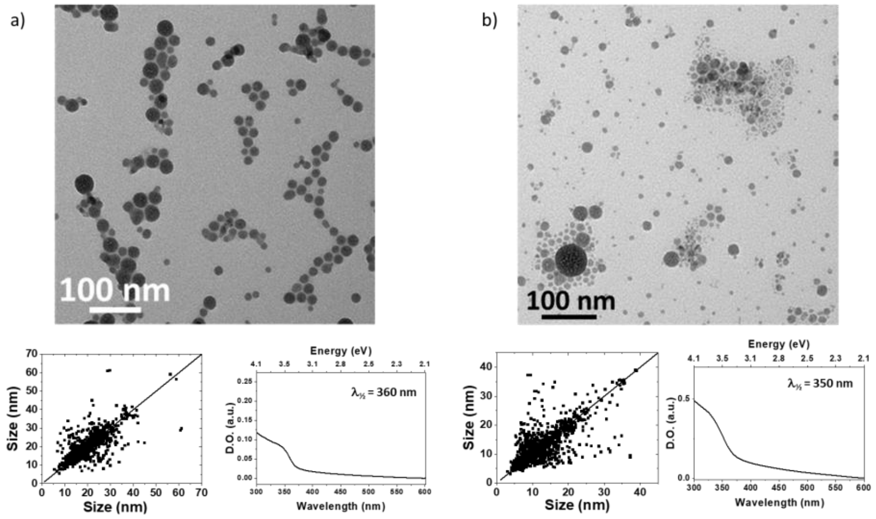

Figure S7. TEM images, associated 2D size plot, and absorption spectra of ZnO NPs formed using the DLRI with the Zn precursor dissolved in a) toluene and b) cyclohexane. For comparison with pentane see Figures 1 and S2. The absorption spectra are similar and characteristic of the nucleation of ZnO NPs of similar size independently of the solvent. However, TEM pictures as well as the associated 2D-size plots evidence that the aggregation state of the NPs varies with mean size of 8 ± 2, 11 ± 8, and 17 ± 12 nm for pentane, cyclohexane, and toluene, respectively.

Preparation of the nanocomposites using DLRI coupled to PE-CVD downstream process:

The Fisher Porter flask containing the liquid solution with precursor(s) was connected to the DLRI device under argon. The DLRI device was plugged to the shower electrode of an asymmetric radiofrequency (13.56 MHz) plasma reactor. Plasma was produced at a working pressure of 0.4 mbar in the steady state and an injected power of 100 W using a Cesar Power Supply coupled with an impedance matchbox connected to the bottom electrode. The reflected power was minimized with less than 1%. While argon enabled to ignite and maintain the discharge, pentane and cyclohexane, dissociated and ionized in the plasma process, were used as the precursors for a Diamond-Like-Carbon (DLC) matrix. Nanocomposite thin films were deposited on silicon substrates for thickness measurements by profilometry or on copper grids for TEM analyses.

The organometallic decomposition methodology prevents any degradation of the injection and PE-CVD equipment. It works in a quasi-continuous mode, with the deposition process at frequencies in the range 0.1-10 Hz range, and few ms aperture.

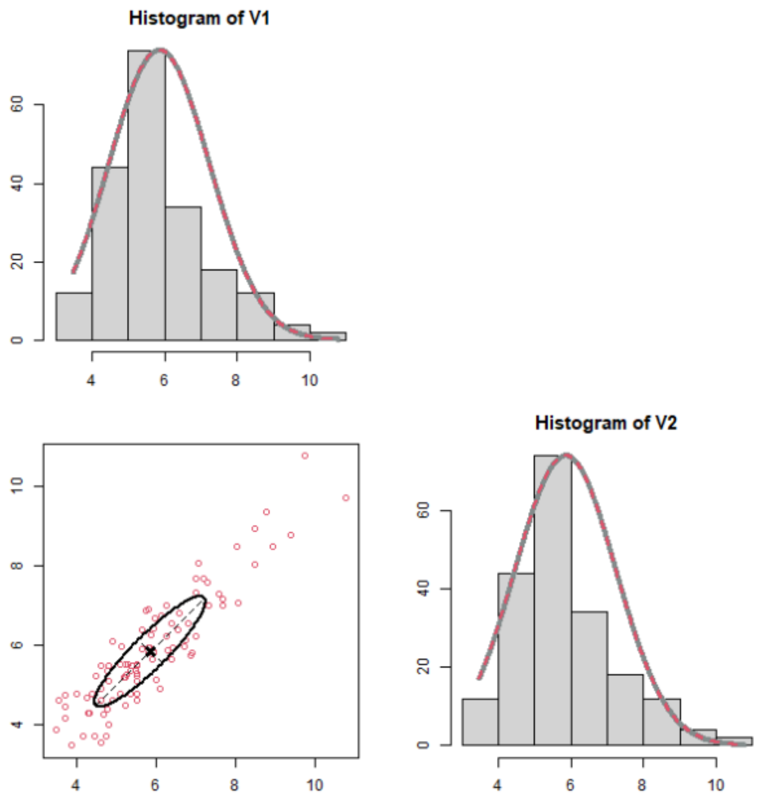

Figure S8. 2D size plot[3] associated to the TEM images of DLC-ZnO nanocomposite obtained by coupling DLRI with PE-CVD. The point cloud lies along the median line, which is characteristic of isotropic NPs. The growth is homogenous in all directions. Multivariate analysis leads to one population with a mean size value of 5.8 ± 1.4 nm.

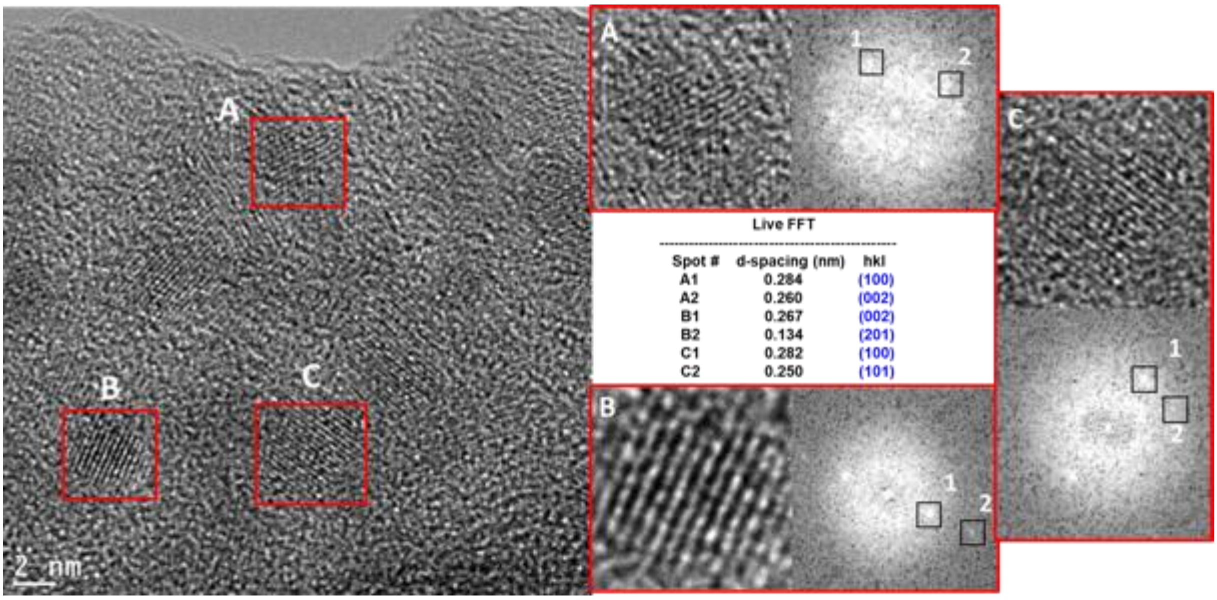

Figure S9. HRTEM of the DLC-ZnO nanocomposite obtained using DLRI coupled with PE-CVD. The distances measured from the Fourier transform are within the accuracy of the measurements, in very good agreement with the (100), (002), (101), and (201) interplane distances anticipated for wurtzite ZnO (0.281, 0.260, 0.247, and 0.136 nm, respectively).

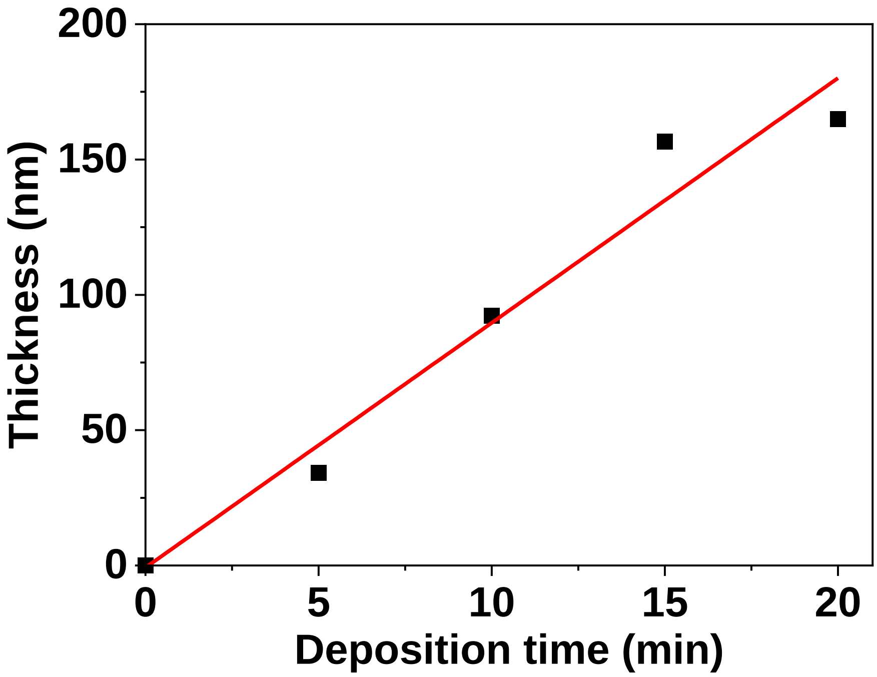

Figure S10. Thickness *vs* deposition time of the nanocomposite films obtained with the DLRI coupled with a low-pressure plasma process.

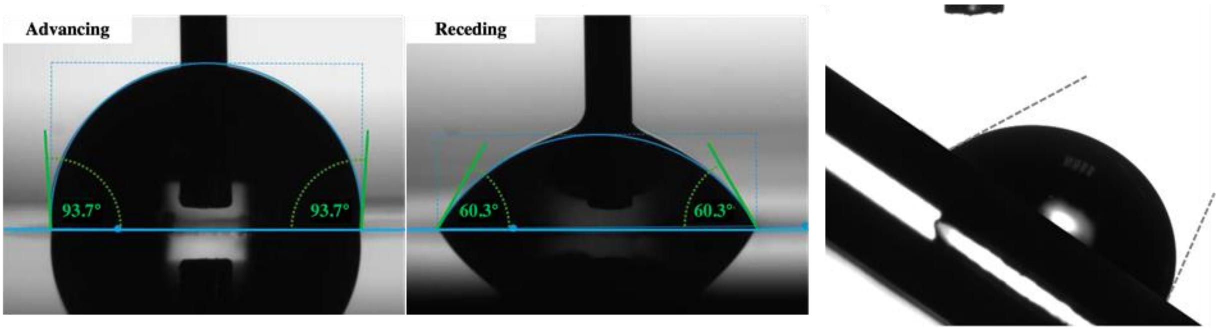

Figure S11. Wettability properties of DLC-ZnO nanocomposite prepared using DLRI coupled to PE-CVD.

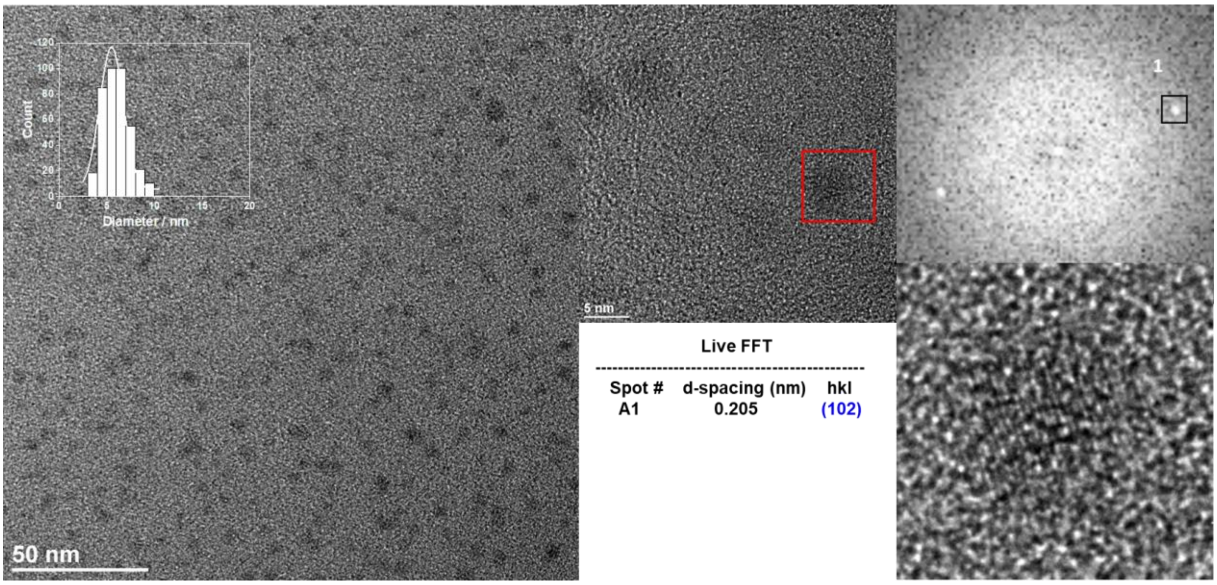

Figure S12. TEM and HRTEM of the SiO_2-ZnO nanocomposite obtained using DLRI coupled to PE-CVD. The distances measured from the Fourier transform are, within the accuracy of the measurements, in very good agreement with the (102) interplane distances anticipated for wurtzite ZnO (0.191 nm).

Preparation of various oxides in DLRI

Preparation of the various oxides in DLR were performed using the same strategy developed for batchwise organometallic NP synthesis.[7,8]

Copper oxide NPs prepared using DLRI

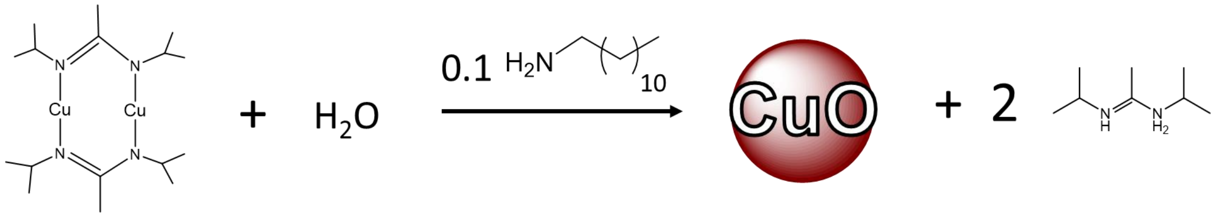

In a glove box, a copper precursor $Cu_2[2,6\text{-(i)}Pr_2C_6H_3N)_2C(H)]_2$[9] (25.7 mg, 0.062 mmole) and dodecylamine (DDA, 2.3 mg, 0.2 equiv., 0.012 mmole) were dissolved in 5 mL of pentane in a dry Fisher Porter tube. DLRI parameters are set to (t_{liq} = 5 ms, t_{out} = 10 ms, Δt = 2 ms, f = 1 Hz). HRTEM images revealed 2.6 ± 0.6 nm crystalline NPs (Figure S13A) with typical copper oxide structure.[7]

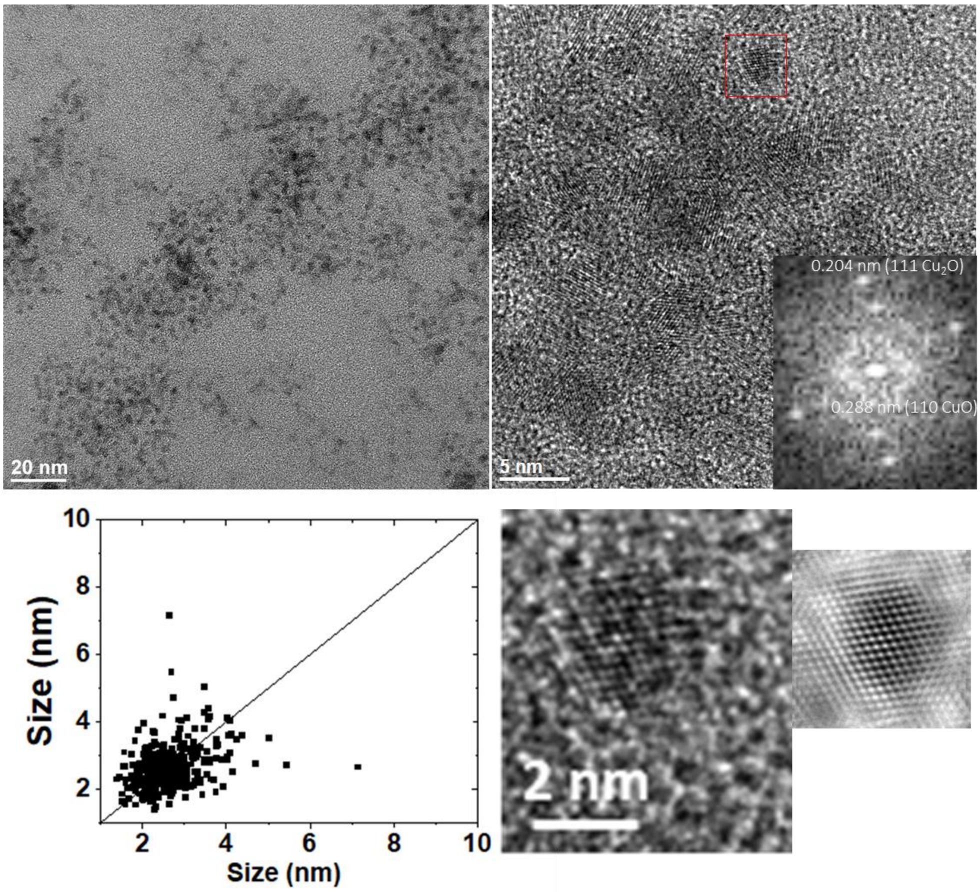

Figure S13A. TEM images and associated 2D size plot of copper oxide NPs prepared in DLRI.

Tin oxide NPs prepared using DLRI

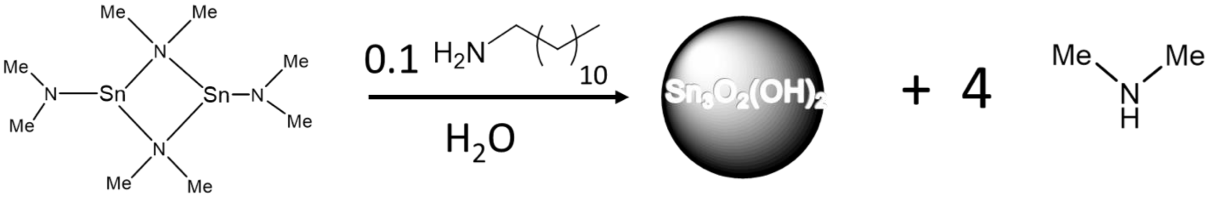

In a glove box, a tin precursor $[Sn(NMe_2)_2]_2$ (77.5 mg, 0.187 mmole, from NanoMeps) was dissolved in 15 mL of pentane in a dry Fisher Porter tube. DLRI parameters are set to (t_{liq} = 5 ms, t_{out} = 10 ms, Δt = 2 ms, f = 1 Hz). HRTEM images revealed ca 2nm size crystalline NPs (Figure S13B) with interatomic distance characteristic of $Sn_3O_2(OH)_2$ phase.[8]

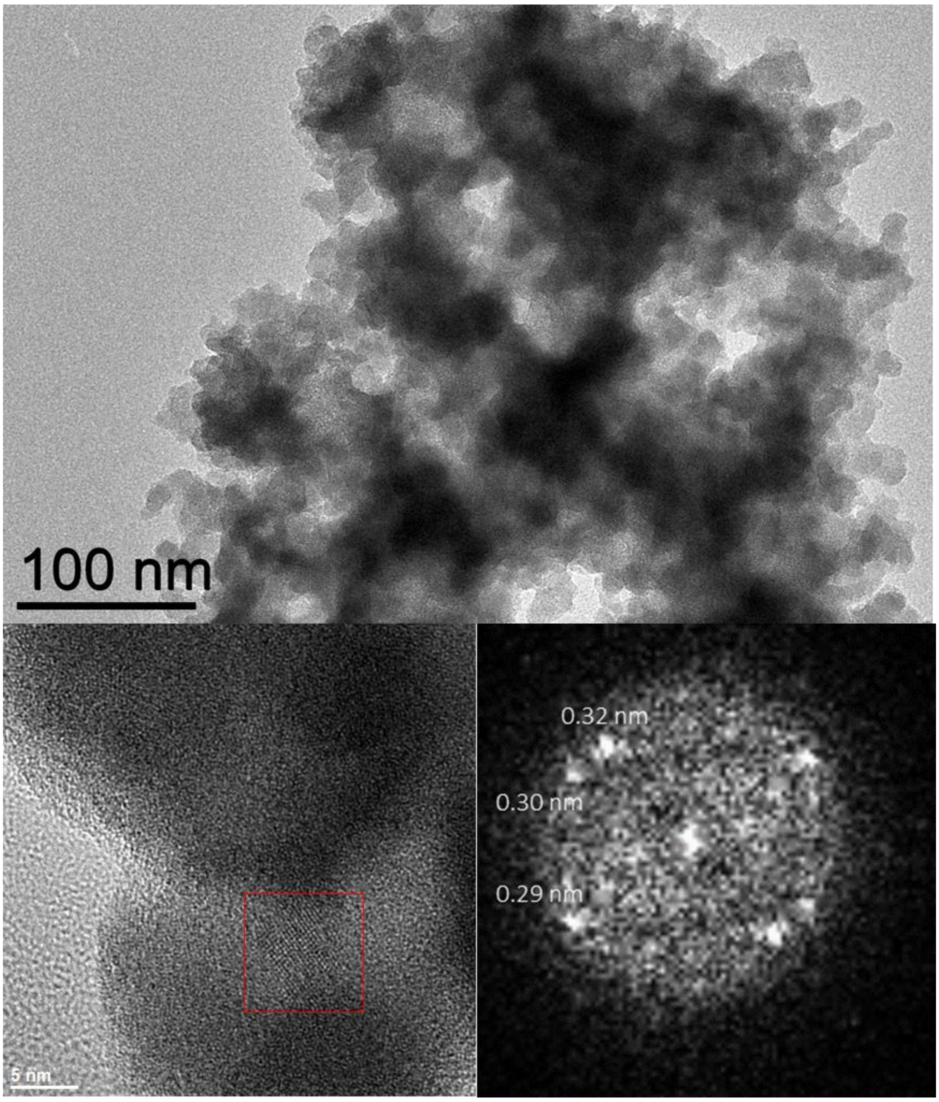

Figure S13B. TEM images and associated 2D size plot of tin oxide NPs prepared in DLRI.

Tungsten oxide NPs prepared using DLRI

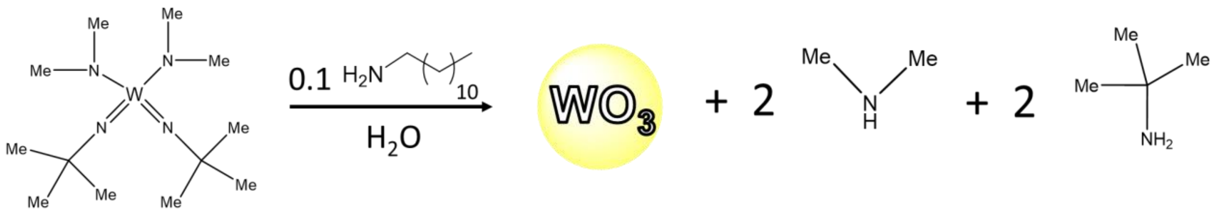

In a glove box, a tungsten precursor $W(NtBu)_2(NHtBu)_2$[10] (58.7 mg, 0.125 mmole) and dodecylamine (DDA, 2.3 mg, 0.1 equiv., 0.012 mmol) were dissolved in 5 mL of pentane in a dry Fisher Porter tube so as to form the liquid phase. DLRI parameters are set to (t_{liq} = 5 ms, t_{out} = 10 ms, Δt = 2 ms, f = 1 Hz). HRTEM images revealed highly crystalline very small NPs (Figure S13C) with an interatomic distance characteristic of a tungsten oxide phase. The accuracy of the measurement does not, however, allow a distinction to be made between the different hydrated phases (WO_3, $WO_3.H_2O$, $WO_3.2H_2O$).

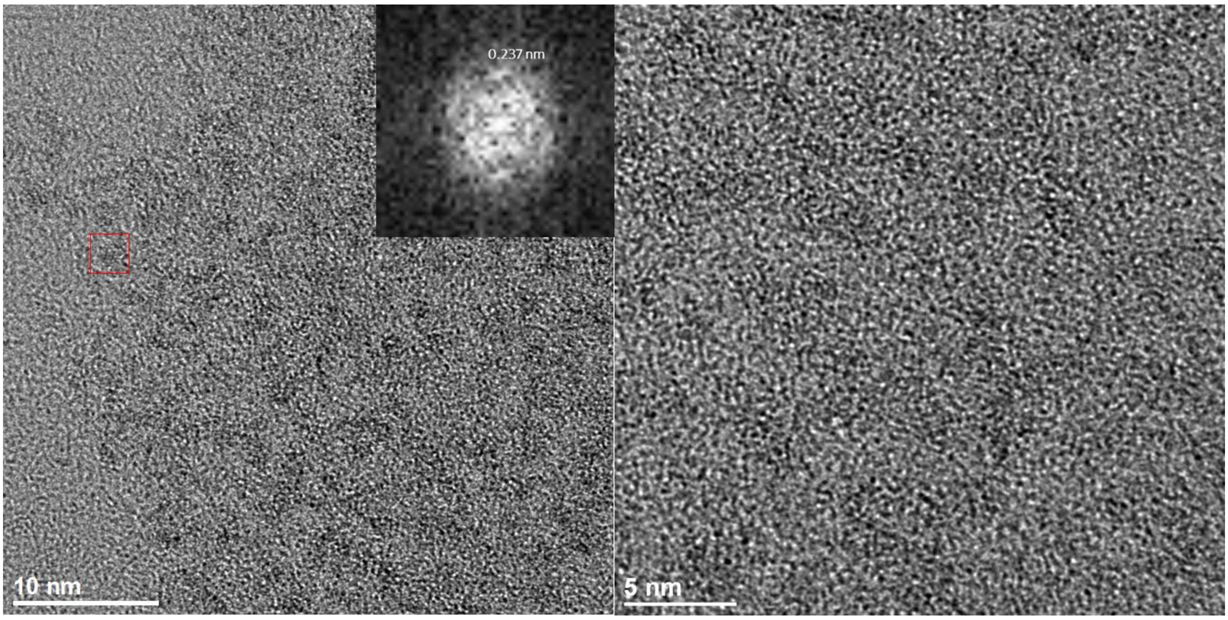

Figure S13C. HRTEM images of tungsten oxide NPs prepared in DLRI.

[1]C.Vahlas, H. Guillon, F. Senocq, B. Caussat, S. Bonnafous, *Gases & Instrumentation* **2009**, 8-11.

[2]M. L. Kahn, A. Glaria, C. Pages, M. Monge, L. Saint Macary, A. Maisonnat, B. Chaudret, *J. Mater. Chem.* **2009**, *19*, 4044-4060.

[3]Z. Zhao, Z. Zheng, C. Roux, C. Delmas, J.-D. Marty, M. L. Kahn, C. Mingotaud, *Chem. Eur. J.*, **2016**, *22*, 12424-12429.

[4]U. Till, L. Gibot, C. Mingotaud, P. Vicendo, M.-P. Rols, M. Gaucher, F. Violleau, A.-F. Mingotaud, *Nanotechnology* **2016,** *27*, 315102.

[5]Difference in size between TEM and DLS measurement is expected for polydisperse samples, as one should bear in mind that DLS favors the detection of large objects compared to small ones, owing to a dependence of the scattered light with the size to the power of 6. Therefore, a solution with a mixture of small and large objects will very likely lead to DLS analyses showing only the large population. This is typically what is observed here where, compared to TEM images, only the large objects are visible in DLS.

[6]M. L. Kahn, M. Monge, V. Colliere, F. Senocq, A. Maisonnat, B. Chaudret, *Adv. Funct. Mater.* **2005,** *15*, 458-468.

[7]J. Jońca, A. Ryzhikov, S. Palussière, J. Esvan, K. Fajerwerg, P. Menini, M.L. Kahn, P. Fau, *ChemPhysChem*, **2017**, *18*, 2658-2665.

[8]J. Jońca, A. Ryzhikov, M. L. Kahn, K. Fajerwerg, A. Chapelle, P. Menini, P. Fau, *Chem. Eur. J.*, **2016**, *22*, 10127-10135.

[9]B. S. Lim, A. Rahtu, J.-S. Park, R. G. Gordon, *Inorg. Chem.*, **2003**, *42*, 7951-7958.

[10]G. M. de Lima, D. J. Duncalf, *Organometallics*, **1999**, *18*, 4884-4886.
